# Gender Differences in the Morphology of the Finger Flexor Pulley System: An Ultrasound-Based Assessment of Recreational Rock Climbers

**DOI:** 10.7759/cureus.83542

**Published:** 2025-05-05

**Authors:** Lance L Lamore, Avery Apostle, Jack Lampert, Amily Tuot, Alexandra Lindgren, Sunny Trivedi, Vy Han

**Affiliations:** 1 Orthopedic Surgery, California University of Science and Medicine, Colton, USA; 2 Plastic Surgery, California University of Science and Medicine, Colton, USA; 3 Anesthesia, California University of Science and Medicine, Colton, USA; 4 Family Medicine, California University of Science and Medicine, Colton, USA; 5 Emergency Medicine, California University of Science and Medicine, Colton, USA; 6 Orthopedic Surgery, Loma Linda University Medical Center, Loma Linda, USA; 7 Medical Education, California University of Science and Medicine, Colton, USA

**Keywords:** a2 pulley, biomechanics, bowstringing, grip strength, rock climbing, tendon

## Abstract

Background

Many rock climbing techniques place tremendous stress on the finger flexor pulley system of the hand and may cause a pulley rupture. A pulley injury can be assessed with ultrasound by measuring an elevated tendon liftoff from the bone during flexion, referred to as the tendon-to-bone distance (TBD). The purpose of this study was to assess gender-specific and experience-related differences in TBD, tendon dimensions, and grip strength among asymptomatic recreational rock climbers to better understand potential anatomical adaptations and injury risks.

Methodology

We conducted a prospective, cross-sectional study at a single indoor climbing facility in San Bernardino County, California, over several days. A total of 65 adult recreational climbers (36 men, 29 women) completed a health and climbing experience survey. A portable ultrasound probe was used to measure TBD, tendon depth, and tendon width at rest and during maximal isometric flexion. Grip strength was measured using a handheld dynamometer. Sex-based differences were analyzed using independent one-tailed t-tests, with significance set at p-values <0.05.

Results

The mean age of the participants was 29.3 ± 7.8 years. On average, women reported a maximum climbing grade of V4, while men reported V6. Men had significantly higher grip strength and tendon cross-sectional area (p < 0.001). The average unflexed TBD was 0.20 cm in women and 0.24 cm in men. During flexion, TBD increased by 0.03 cm in women and 0.05 cm in men, with no significant difference (p = 0.07).

Conclusions

In this study, women exhibited significantly smaller tendon cross-sectional area and lower grip strength compared to men. These anatomical differences warrant further research to explore implications for climbing performance and injury risk.

## Introduction

Finger pulley injuries, especially of the A2 and A4 pulleys, are among the most common injuries affecting climbers due to repetitive high-load finger movements required in sport climbing [1,2]. This study aims to evaluate whether anatomical differences in tendon-to-bone distance (TBD), tendon size, and grip strength exist between male and female recreational climbers, and whether climbing experience is associated with these adaptations. These variables have been implicated in the mechanical loading and structural support of the pulley system and may influence the risk of A2 pulley injury through their role in tendon bowstringing and force transmission during flexion [3-6].

In the last decade, rock climbing has seen a dramatic surge in popularity, with 4.4% of the US population (approximately 7.7 million individuals) participating in indoor climbing, a trend reflected in the sport’s inclusion in the 2020 Tokyo Olympics [7]. As of 2014, there were 7.7 million participants within the United States, and a documented 6.6% growth between 2021 and 2022 [8]. Several common injuries are reported among climbers. The upper extremity is most commonly affected by climbing injuries. A survey of 357 climbers reported that 321 (90%) had sustained an upper extremity injury [1]. A separate study surveying 237 climbers reporting their history of injury revealed that the most common types of injury were to the hand and elbow (99/237, 41.9%), followed by foot and ankle (47/237, 19.9%), shoulder (41/237, 17.3%), knee (17/237, 7.4%), spine (9/237, 3.7%), hip (7/237, 2.8%), and others (16/237, 6.9%) [9]. These findings align with emergency department data from the National Electronic Injury Surveillance System, which also identifies lower extremities as common sites of climbing injuries (47%) out of 34,785 rock climbing injuries, with fractures, sprains, and strains as the most frequently reported injuries [10]. Among upper extremity injuries, the majority involve the hand and wrist, particularly the fingers, with digital flexor sheath ruptures, tendinopathies, and strains being the most common types [1,2]. Consistent with these findings, overuse injuries have been reported as the predominant injury type among sport climbers, likely due to repetitive stress on tissues during frequent climbing sessions [11,12].

One of the most commonly observed upper extremity injuries among rock climbers is a traumatic rupture of the digital flexor pulley system [13]. The digital flexor pulley system consists of a set of fibrous bands that encapsulate the flexor tendons to ensure close proximity of the tendon and phalanx during flexion, thus enabling an efficient transfer of muscular force into mobilization of the interphalangeal joint [14]. The pulley system is composed of five annular, or ring-shaped, pulleys (A1-A5) and three cruciate (C1-C3) pulleys [15]. The A2 and A4 are at the mid-section of the proximal and middle phalanges, respectively, and are the most commonly affected pulleys in rock climbers. Insufficiency of these structures is most responsible for tendon “bowstringing,” which describes a phenomenon whereby the tendon lifts off from the bone during flexion [3]. Recent injury trends suggest that A4 pulley injuries may now be occurring more frequently than A2 injuries, potentially related to evolving climbing techniques and higher intensity moves [11]. Digits three and four are the most commonly affected in rock climbers [15].

Traumatic rupture of the A2 flexor pulley is a consequence of the “crimp” grip position in which the proximal interphalangeal joint is flexed and the distal interphalangeal joint is hyperextended against excessive force (>400 N) [4,16]. The biomechanical interaction between flexor tendons and pulleys has been described in detail, emphasizing that friction and mechanical stress play a substantial role in pulley injuries [5]. While bowstringing of the tendon was initially thought to pathologically affect grip strength and efficient movement of the finger alone, previous literature has pointed to the possibility that mild bowstringing can develop as an asymptomatic physiological advantage among experienced climbers [6]. To distinguish between pathological or physiological mechanisms, an injury threshold of 0.20 cm [9] for tendon liftoff has been proposed [6]. Pain due to this injury is described as an audible pop and an accompanying tearing sensation, followed by chronic pain, discomfort, and deficits in grip strength and finger dexterity [4,17]. Finger flexor pulley system ruptures are most commonly diagnosed with ultrasound, which offers an opportunity for a dynamic view of the tendon from flexion to relaxation [18]. Ultrasound is widely considered equivalent or superior to MRI in diagnosing pulley ruptures due to its accessibility, lower cost, and dynamic visualization capabilities [19-22]. By applying resistance during finger flexion and measuring the TBD distance at maximal flexion, clinicians can assess the degree of bowstringing 6].

According to a nationally representative survey of 20,069 individuals conducted by the Outdoor Foundation, a nonprofit organization that tracks outdoor recreation trends in the United States, females comprised 51% of rock climbers in 2019 [23]. Recent literature has highlighted gender differences in climbing injuries, emphasizing the need to document gender-specific risks and injury profiles to better understand injury mechanisms among female climbers [24,25]. It has been observed that female rock climbers are more likely to report an upper extremity injury and pursue surgical intervention [1]. Thus, our study aims to include climbers of all genders and experience levels, expanding the literature to a demographic representative of the growing rock climbing community. The purpose of this study was to evaluate whether anatomical differences in TBD, tendon size, and grip strength exist between male and female recreational climbers, and whether climbing experience is associated with these adaptations.

## Materials and methods

Study design and setting

This prospective, observational, cross-sectional study recruited 65 recreational rock climbers from a rock climbing facility located in San Bernardino County, California. Institutional Review Board approval for the use of human subjects was obtained from the California University of Science and Medicine (approval number: HS-2023-34). In this prospective study, the primary outcome measures, including TBD, tendon depth, tendon width, and grip strength, were established before data collection.

Participant selection and climbing experience

Climbing proficiency was assessed using the American V-scale (also known as the Hueco Scale), a grading system commonly used in North America to rate bouldering difficulty. The scale ranges from V0 (easiest) to V17 (most difficult), with higher values indicating more advanced skill levels. Eligibility required participants to be at least 18 years old and capable of completing a minimum of a V1-grade climbing route, which allowed for the inclusion of individuals spanning a wide spectrum of climbing abilities, from beginner to advanced. Exclusion criteria comprised individuals actively experiencing pain during climbing within the designated anatomical location of the study. All participants provided electronic informed consent.

A survey was administered through Qualtrics XM (Qualtrics, Provo, UT) to gather demographic information, prior climbing experience, and other pertinent history from participants. The survey included questions regarding medical history, including prior musculoskeletal injuries, systemic illnesses, or comorbidities. Participants who reported systemic illnesses, ongoing pain during climbing, or a history of traumatic or surgical injuries to the fingers, hand, or wrist were excluded to minimize confounding effects on tendon morphology and grip strength. Climbing experience was operationalized based on the number of self-reported years of climbing and maximum climbing proficiency, reported using the American V-grade scale. Experience-related differences were examined by analyzing trends in TBD across varying years of climbing and climbing skill level. Climbing experience was treated as a continuous variable for correlation analyses and binned into V-grade categories for group comparisons.

Grip strength protocol

**Figure 1:**
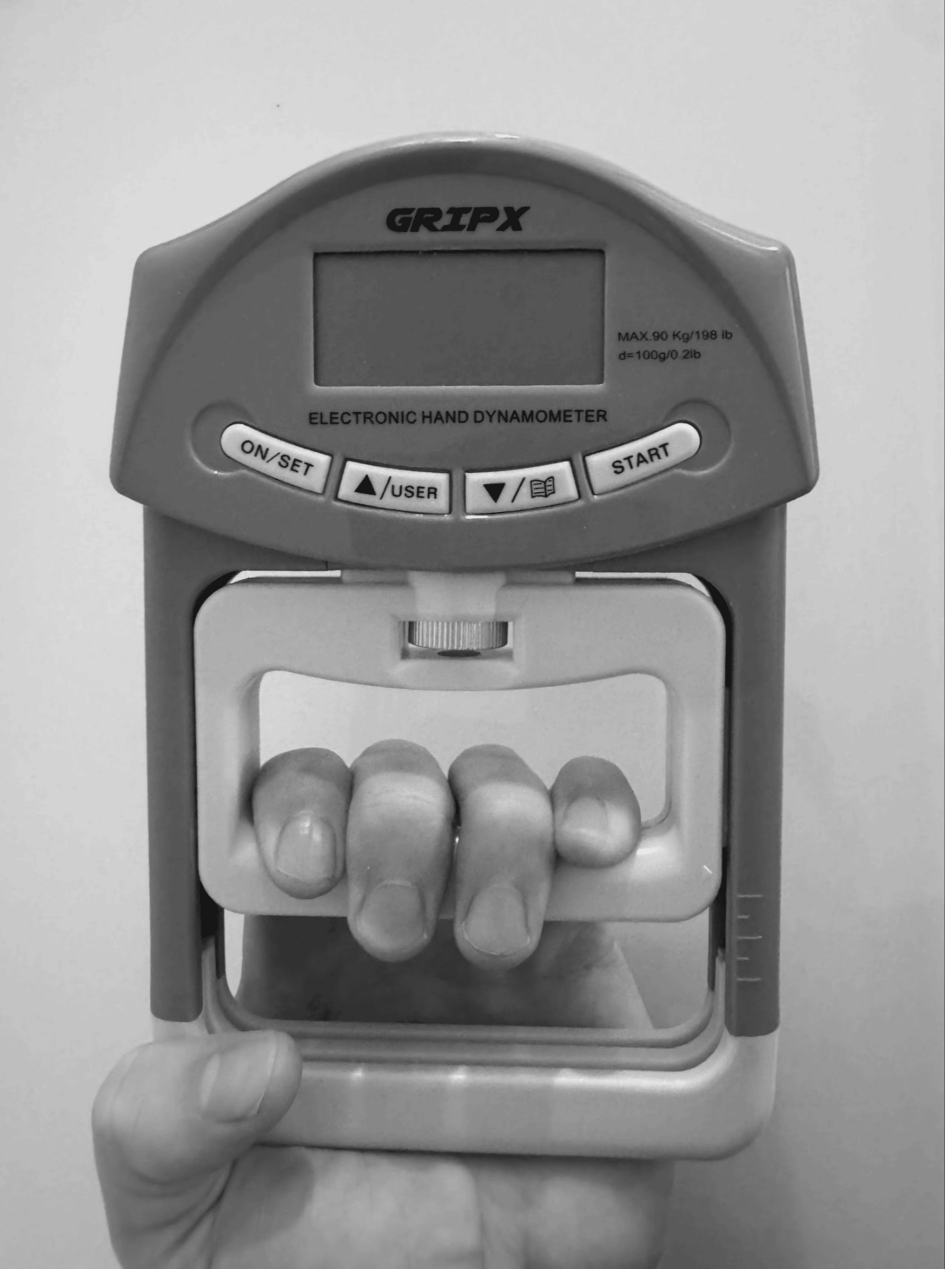
Dynamometer used in the measurement of the grip strength.

Grip strength was measured using a handheld dynamometer (Grip X) with the participant standing, shoulder and elbow held in the position most comfortable to the patient, forearm in a neutral position, and wrist positioned between 0-30 degrees extension. The dynamometer is shown in Figure 1. Participants were instructed to squeeze maximally for a few seconds below their pain threshold. Two trials were conducted with approximately 30 seconds of rest in between, and the higher of the two values was recorded and used for analysis.

Ultrasound imaging and TBD measurement

**Figure 2:**
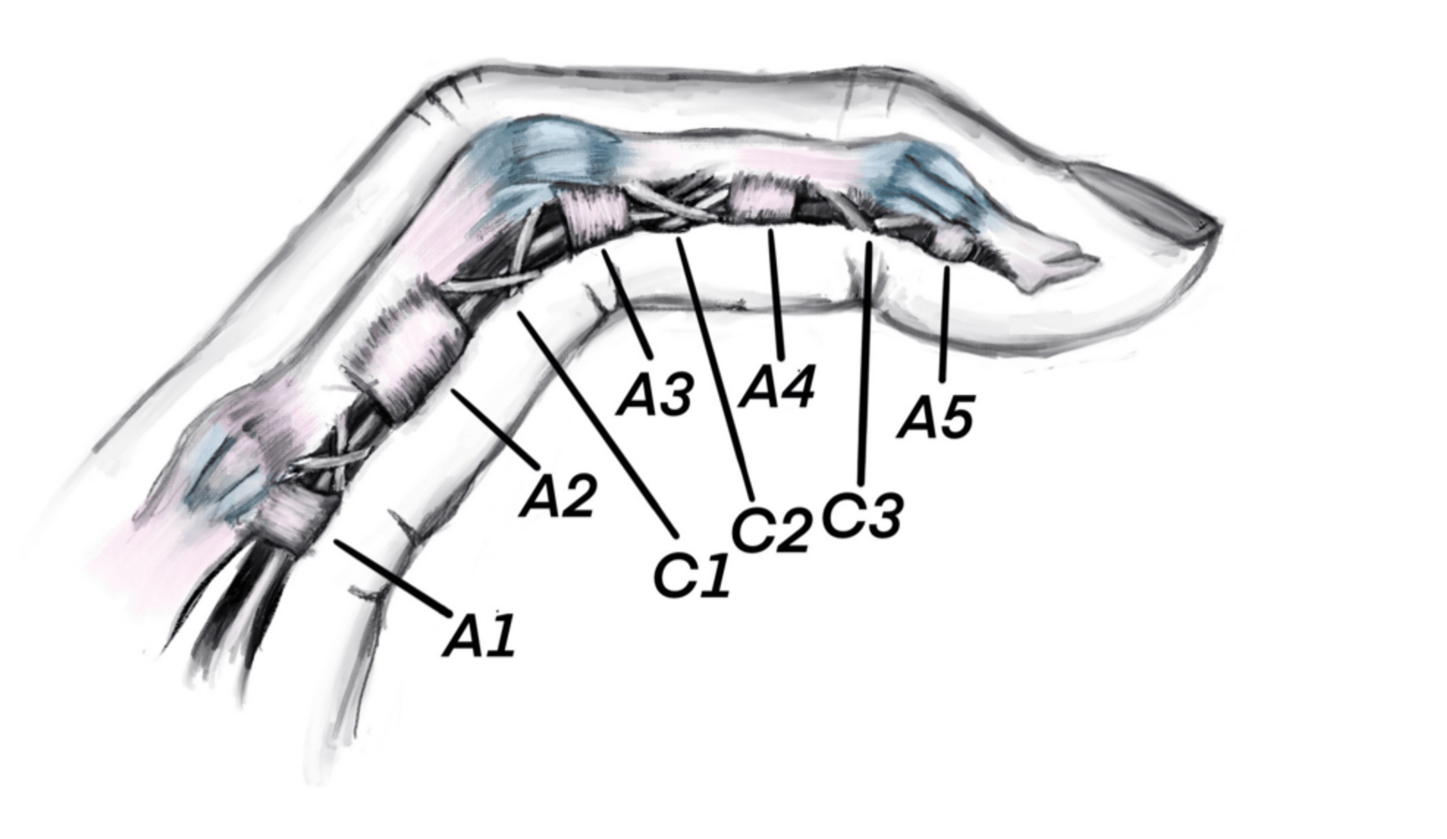
Anatomy of the finger flexor pulley system demonstrating the five annular and three cruciate pulleys that stabilize the position of the flexor tendon sheath as it courses its way to the distal phalanx. Redrawn by authors based on Horsch (2008) [26].

A Butterfly iQ+ (Butterfly, Burlington, MA) portable ultrasound device was utilized to measure the dominant index finger’s proximal phalanx TBD, tendon depth, and tendon width [6]. Figure 2 provides a schematic illustration of the finger flexor pulley system, including the flexor tendons, phalanges, and annular pulleys.

Participants were seated comfortably during the ultrasound examination, with the dominant forearm resting on a flat surface and the wrist maintained in a supinated position. The index finger pad was stabilized beneath a secure, non-elastic band to facilitate consistent force application during tendon loading. All ultrasound measurements were acquired at the midpoint of the proximal phalanx of the index finger.

The phalangeal midpoint was first determined externally using a standard ruler to measure the total length of the proximal phalanx. This value was then halved, and the corresponding point was located on ultrasound using the device’s internal caliper tool, measured from the distal end of the phalanx.

**Figure 3:**
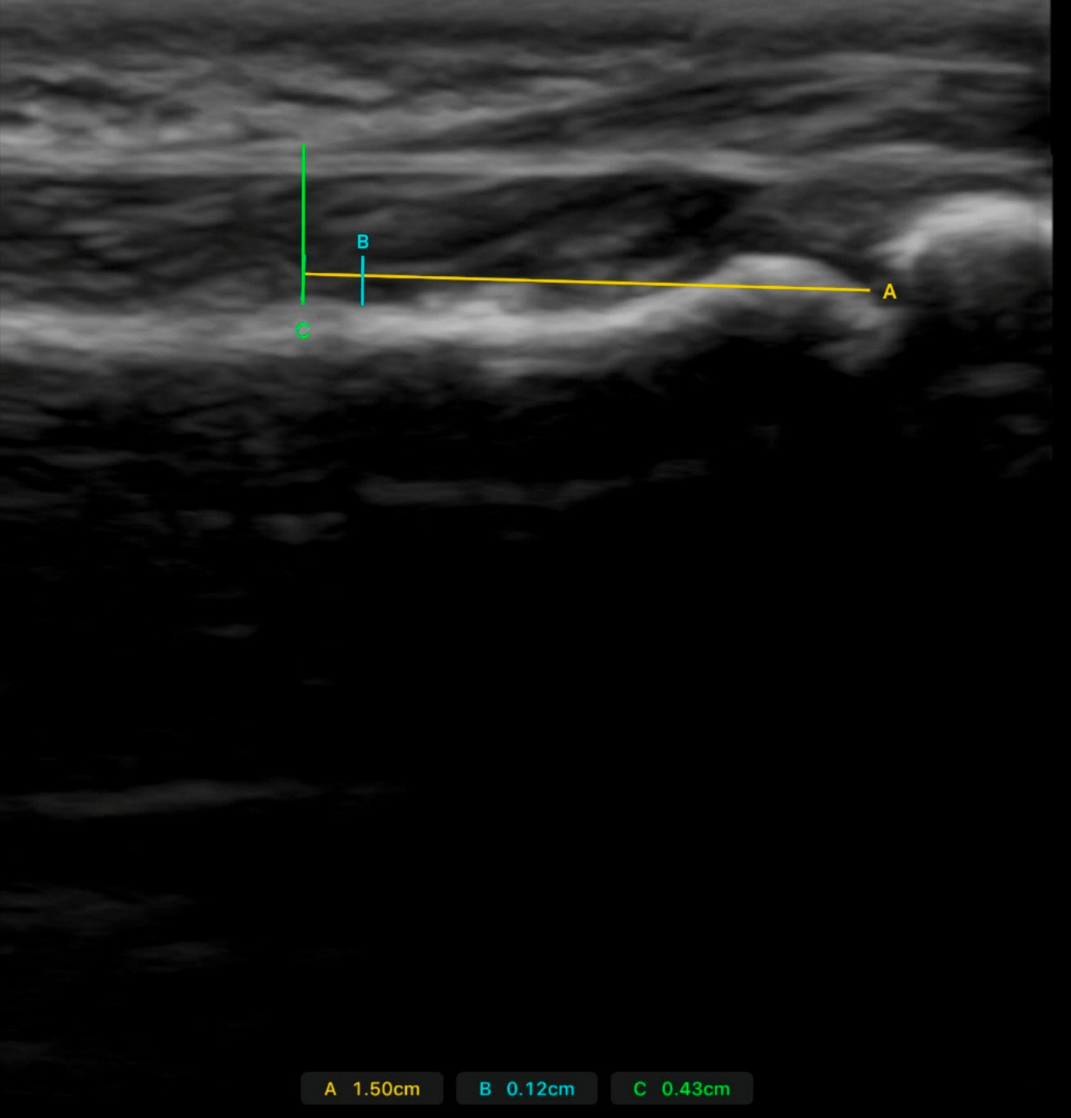
A longitudinal ultrasound view of the finger flexor tendon. Distance A (yellow line) represents the distance to the midpoint of the proximal phalanx, distance B (blue line) represents the distance between the tendon and the bone, and distance C (green line) represents the distance from the superficial border of the tendon sheath to the bone.

**Figure 4:**
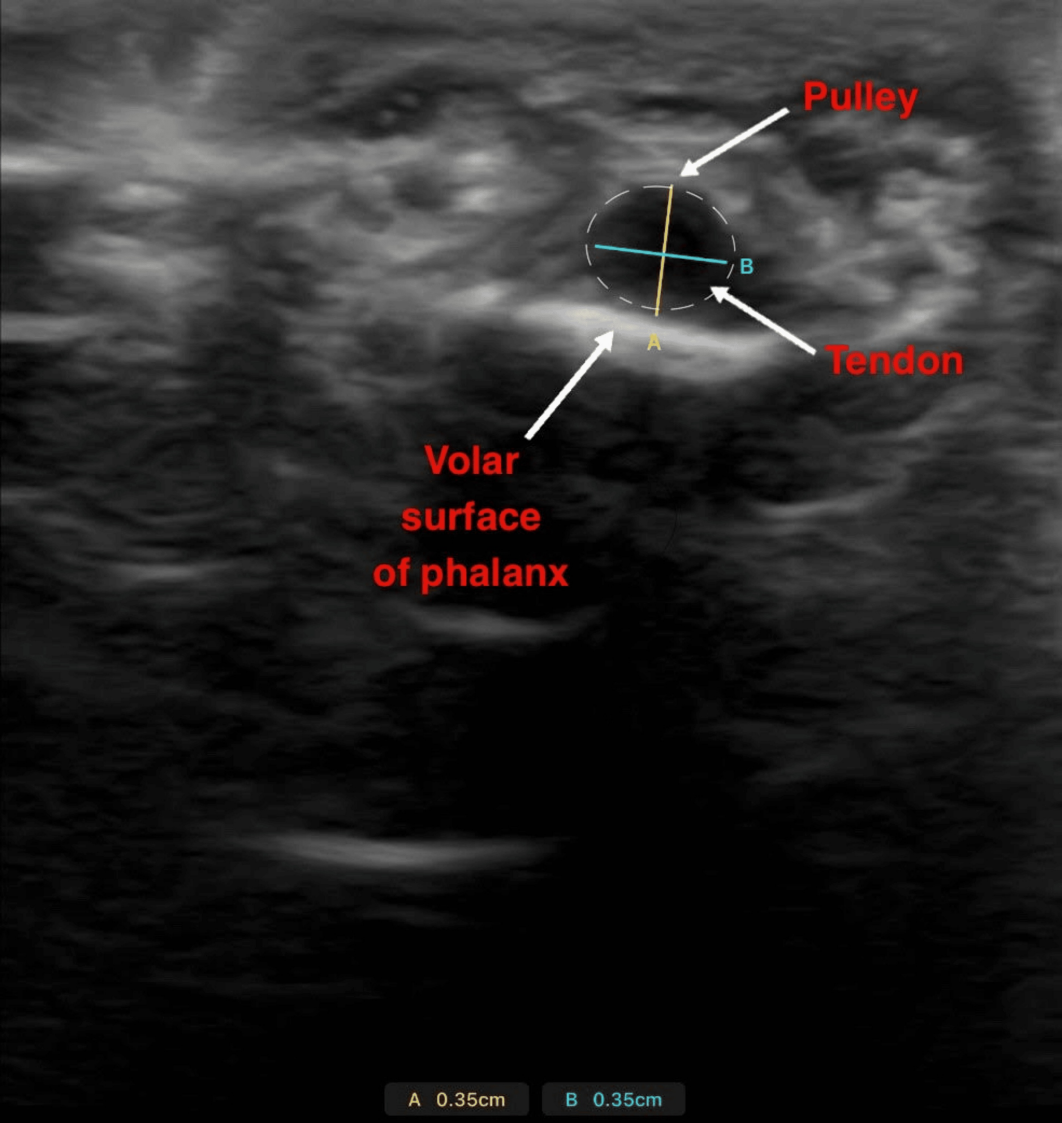
A cross-sectional ultrasound view of the finger flexor tendon. The tendon border is illustrated by the dashed oval. Distance A (yellow line) captures the tendon depth, while distance B (blue line) captures the tendon width. Surrounding structures are labeled.

TBD was recorded in the longitudinal plane under a relaxed state and during maximal isometric flexion, during which participants were instructed to apply force against the stabilization band beneath their pain threshold while maintaining static joint angles. After completion of the TBD measurements, the ultrasound probe was repositioned in the transverse plane to capture tendon depth and width from a cross-sectional view of the flexor tendon sheath. These views are demonstrated in Figure 3 and Figure 4. The method was adapted from previous tendon-loading protocols [6].

Statistical analysis

Continuous variables were reported as mean ± standard deviation. Data normality was assessed using the Shapiro-Wilk test. Statistical analyses were conducted utilizing one-tailed independent t-tests to compare male versus female climbers for TBD, tendon depth and width, and grip strength, with significance set at p-values <0.05. In tables, statistically significant results were annotated using symbols (* for p < 0.05). No a priori sample size calculation was performed given the exploratory nature of this study; however, sample size was guided by feasibility and prior similar studies in climbing biomechanics [6].

## Results

Our study enrolled 65 recreational climbers, with 36 male participants and 29 female participants. Demographic data, climbing experience, and climbing skill levels are summarized in Table 1. While most variables were normally distributed (p > 0.05), the level of climbing and years of climbing showed mild deviation. However, given the sample size and robustness of the t-test, we proceeded with parametric testing. Male climbers had significantly higher average body weight, height, and body mass index (BMI) compared to female climbers (Table 1). Descriptive statistics are reported as mean ± standard deviation for continuous variables, with statistical significance defined as p < 0.05.

**Table 1:** Demographic data of male and female recreational rock climbers. Data are presented as mean ± SD. Statistical comparisons between male and female climbers were conducted using independent one-tailed t-tests. The t-value for each test is shown in the second to last column, followed by the corresponding p-value. A p-value < 0.05 was considered statistically significant. ^a^: American V-grade scale; *: p < 0.05

	Male	Female	t-value	P-value
	Mean ± SD	Mean ± SD		
Number	36	29		
Age (years)	30.4 ± 8.3	29.0 ± 6.5	0.756	0.2278
Weight (kg)	72.2 ± 9.79	56.31 ± 12.1	6.856	<0.0001*
Height (cm)	175 ± 8.11	163 ± 6.76	7.946	<0.0001^*^
Body mass index (kg/m²)	23.43 ± 2.23	21.84 ± 2.41	2.856	0.0040^*^
Level of climbing^a^	6.03 ± 1.96	4.17 ± 1.67	4.390	<0.0001^*^
Years of climbing	5.90 ± 7.41	2.87 ± 3.5	2.154	0.0243^*^

Male participants had significantly greater climbing experience than female participants, with mean years of experience approximately twice as high (p = 0.0243). Male climbers also demonstrated significantly higher climbing skill levels, averaging nearly two V grades higher compared to female climbers (p < 0.0001). Primary ultrasound measurements (Table 2) indicated several significant anatomical differences.

**Table 2:** Measurements of male and female recreational rock climbers. Data are presented as mean ± SD. Statistical comparisons between male and female climbers were conducted using independent one-tailed t-tests. The t-value for each test is shown in the second-to-last column, followed by the corresponding p-value. A p-value <0.05 was considered statistically significant. TBD: tendon-to-bone distance; *: p < 0.05.

	Male (n = 36)	Female (n = 29)	t-value	P-value
	Mean ± SD	Mean ± SD		
Proximal phalanx length (cm)	5.63 ± 0.44	5.28 ± 0.28 cm	3.935	0.0003^*^
TBD (cm)				
Relaxed	0.19 ± 0.051	0.17 ± 0.059	1.745	0.0467*
Flexed	0.24 ± 0.062	0.20 ± 0.05	3.306	0.0013*
Difference	0.05 ± 0.062	0.03 ± 0.068	1.465	0.0775
Cross-section (cm)				
Depth	0.42 ± 0.076	0.34 ± 0.05	4.118	0.0002*
Width	0.57 ± 0.14	0.48 ± 0.09	3.117	0.0021*
Grip strength (lbs)	107.0 ± 27.0	63.8 ± 10.6	8.795	<0.0001*

Male participants exhibited significantly longer proximal phalanx lengths compared to females (p = 0.0003). During relaxation, the TBD was approximately 10% greater in male participants compared to female participants (p = 0.0467). During flexion, male climbers displayed significantly greater TBD distances, approximately 20% greater than female climbers (p = 0.0013). The difference in TBD between relaxed and flexed states was greater in male participants, although this difference did not reach statistical significance (p = 0.077).

Male climbers exhibited significantly greater tendon cross-sectional dimensions, with larger tendon depth (p = 0.0001) and tendon width (p = 0.0021). Grip strength measurements were also significantly higher among male participants, averaging approximately 107 lbs compared to roughly 64 lbs for female participants (p < 0.0001).

**Figure 5:**
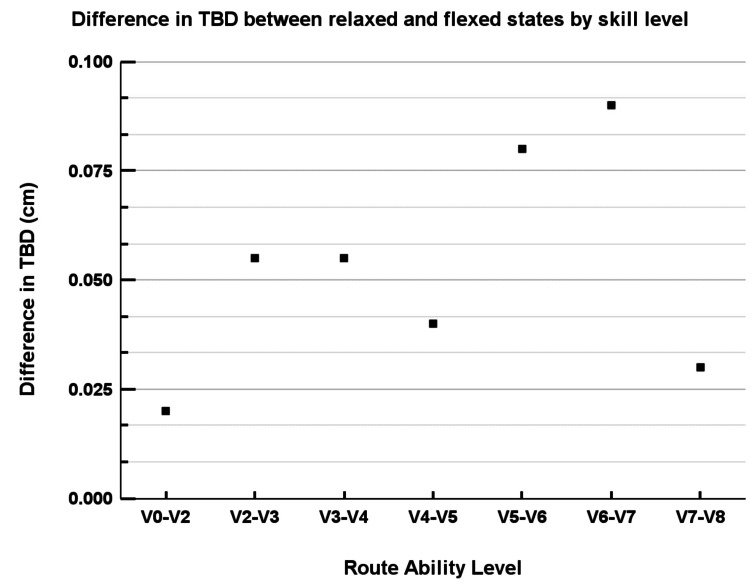
Difference in TBD by skill level. The graph depicts the difference in tendon-to-bone distance (TBD) between relaxed and flexed states across various rock climbing route ability levels. The y-axis represents the difference in TBD in centimeters, while the x-axis categorizes the climbers by their typical American V grade climbing scale. To ensure meaningful subgroup comparisons, only climbing grades with at least three participants were included in stratified analyses.

Figure 5 shows mean differences in TBD between relaxed and flexed states aggregated across climbing skill categories. Climbers with higher climbing ability (higher V-grade categories) exhibited greater average TBD differences, suggesting a potential adaptive response associated with increased climbing proficiency.

**Figure 6:**
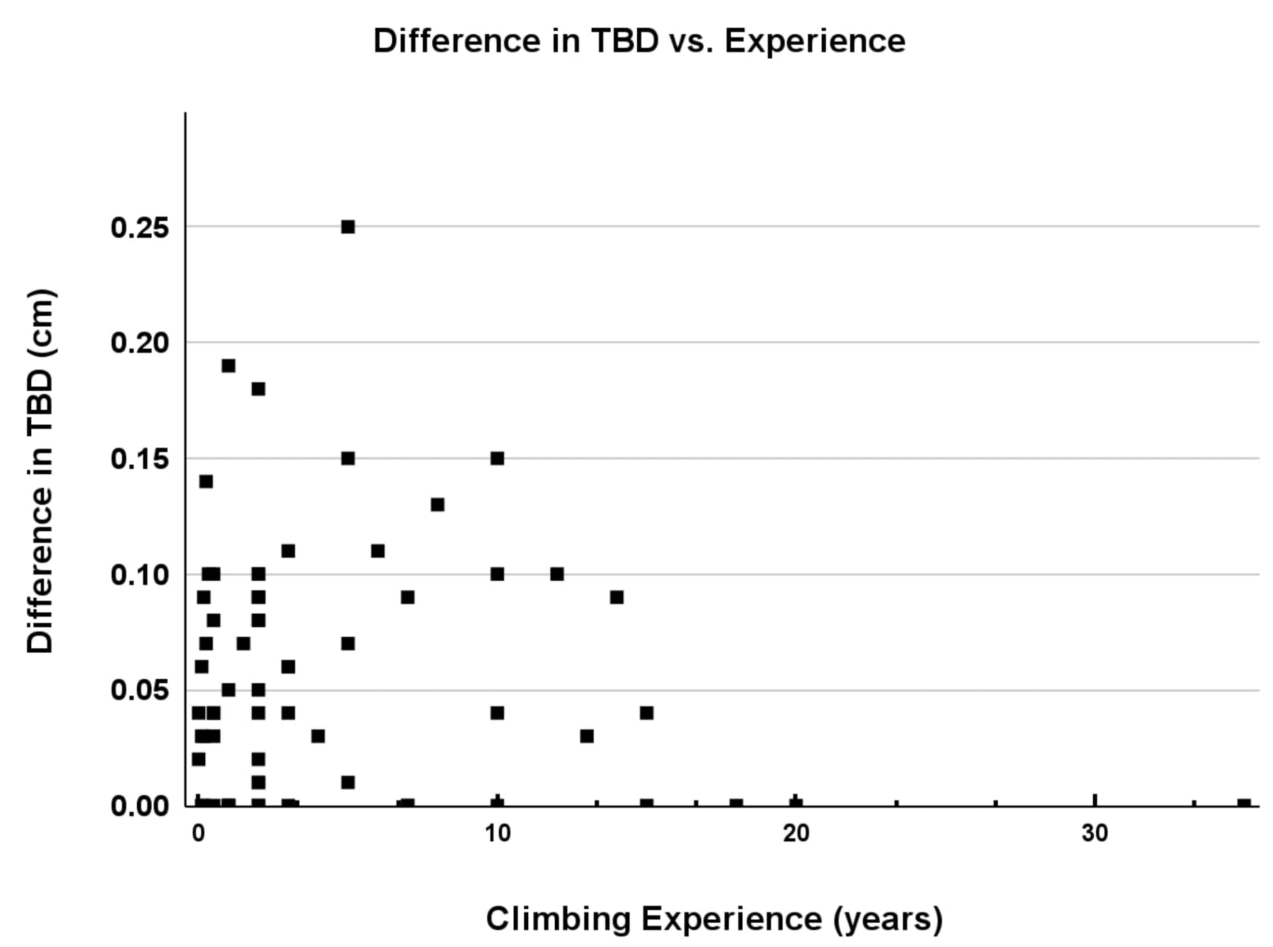
Difference in TBD by experience. Scatter plot showing difference in tendon-to-bone distance (TBD) measurements during flexion across self-reported years of experience. All individual data points are shown.

Figure 6 depicts the association between the difference in TBD and years of climbing experience, indicating a trend where climbers with more experience generally exhibited greater TBD differences, which is consistent with adaptive anatomical changes in response to repetitive stress.

**Figure 7:**
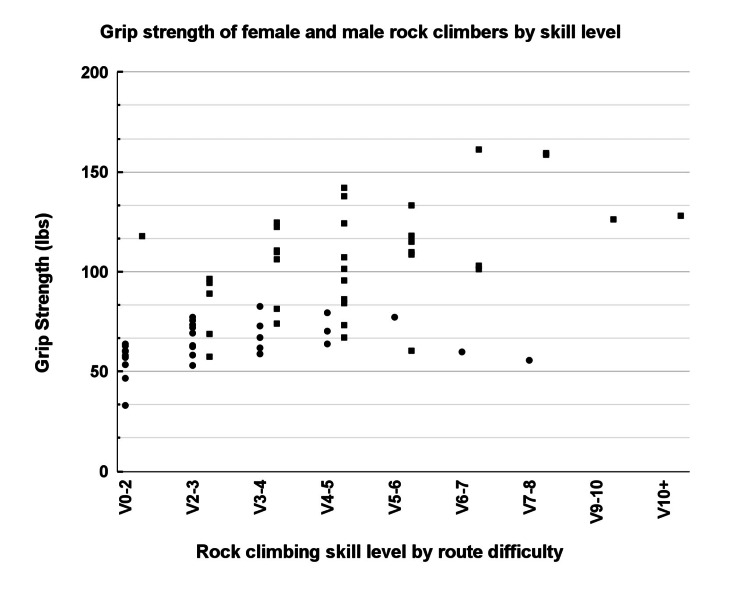
Grip strength by skill level. The graph compares grip strength across female (circles) and male (squares) rock climbers of varying skill levels. The y-axis represents the grip strength measurement in (lbs), while the x-axis indicates the self-reported rock climbing skill by route difficulty, ranging from V0-2 to V10+. All individual data points are shown.

Figure 7 compares grip strength measurements between male and female climbers across various skill levels. Although male climbers consistently demonstrated significantly greater grip strength, increases in grip strength with higher skill levels appeared relatively modest. This observation may suggest that improvements in climbing performance at higher skill levels rely more heavily on technical efficiency than solely on muscular strength gains.

## Discussion

The finger flexor pulley system is uniquely challenged during rock climbing, facing significant frictional, compressive, and tensile forces [18]. The repetitive loading inherent to climbing, particularly in positions such as the crimp grip, can induce anatomical adaptations in the tendon and pulley system, including increased tendon volume and strength [27,28]. Differences in pulley stress between concentric and eccentric loading suggest that eccentric loading may particularly increase injury susceptibility [11]. These anatomical adaptations may reflect a response to chronic repeated subclinical stresses or from healed injuries, resulting in increased tendon laxity [29].

Our study identified statistically significant anatomical differences in TBD, tendon dimensions, and grip strength between male and female climbers. While anatomical differences alone do not confirm distinct injury patterns, these observed differences may contribute to potential variations in injury risk and underscore the importance of considering sex-specific factors in injury prevention strategies. While lower body weight and BMI in females may decrease overall mechanical load on tendons and pulleys, smaller tendon size and lower grip strength may compromise structural integrity under high stress, potentially elevating injury risk [22,24]. Previous studies have highlighted substantial biomechanical loads experienced by the A2 pulley during climbing, particularly in crimp grips requiring support from fewer fingers [30,31]. Given that pulley adaptation may be necessary to withstand these high forces [16,32], gender-specific anatomical characteristics should be considered in injury prevention protocols.

As illustrated in our results (Figures 5, 6), climbers with greater climbing ability and more climbing experience exhibited increased TBD differences between relaxed and flexed states. These findings suggest that repetitive mechanical stress may stimulate adaptive anatomical changes in the tendon-pulley system. Previous research has similarly reported morphological adaptations in advanced climbers, including increased laxity and thickening of the pulleys, potentially contributing to improved mechanical tolerance under load [3,6]. Thus, larger TBD differences among higher-level climbers likely represent a beneficial adaptive response rather than pathology.

Our results indicated substantial differences in grip strength between male and female climbers, and grip strength was positively associated with climbing experience and skill level. However, relatively modest increases in grip strength among higher-skilled climbers suggest that climbing performance may rely more heavily on technique and efficiency rather than strength alone. While technique may help distribute biomechanical loads effectively, reliance on technical skill over strength may potentially increase vulnerability to injury if unexpected forces exceed tissue tolerance. Future studies should consider incorporating body weight and broader measures of physical activity to evaluate their relationship with grip strength, which may offer additional insights into musculoskeletal performance and injury risk among climbers.

Limitations

While our sample included climbers with a range of body sizes, ages, and experience levels, we did not control for anthropometric covariates such as BMI or forearm circumference, which may influence tendon morphology and grip strength. Furthermore, variability in distribution could influence group comparisons. These sources of variability may limit the strength of our group comparisons and should be addressed in future studies with matched or stratified samples.

Ultrasound imaging was performed by two operators with training in musculoskeletal ultrasound. Although both followed a standardized protocol and all measurements were reviewed and verified by the study team to enhance consistency, the absence of formal certification and lack of inter-rater and intra-rater reliability testing may introduce variability.

Additionally, our study did not include clinical injury assessments or symptomatic data, limiting our ability to definitively distinguish adaptive tendon responses from subclinical or pathological changes [3]. Longitudinal studies incorporating symptomatic and asymptomatic climbers are warranted to better determine whether observed anatomical differences translate into differential injury risk.

## Conclusions

Our findings demonstrate anatomical differences in finger flexor pulley tendon morphology and grip strength associated with gender and climbing experience among recreational rock climbers. Our data underscore the potential relevance of gender-specific and experience-related factors in understanding finger flexor pulley biomechanics. Further research across genders is needed to determine whether these anatomical differences can inform improved injury prevention, detection, and rehabilitation strategies, ultimately promoting safer climbing practices for both men and women.
